# Supplementary figures and images for: Elevated phosphorylation of EGFR in NSCLC due to mutations in PTPRH
Source: PLoS Genet. 2022 Sep 2;18(9):e1010362. doi: 10.1371/journal.pgen.1010362 (PMC9477422; doi:10.1371/journal.pgen.1010362)

Swiatnicki et al, Supplemental Figure 1

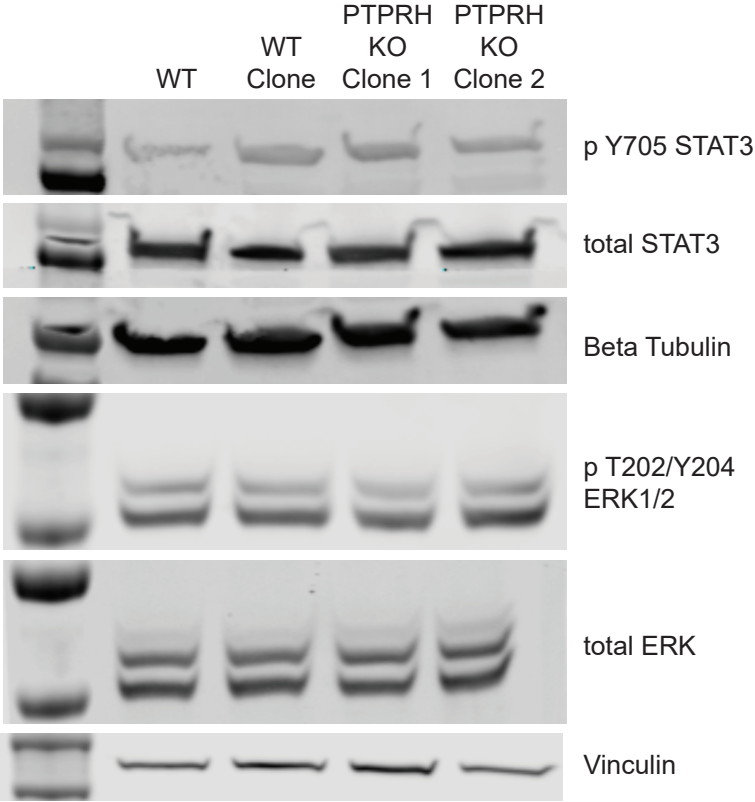

Supplement: S1 Fig — (PDF) [file pgen.1010362.s001.pdf]

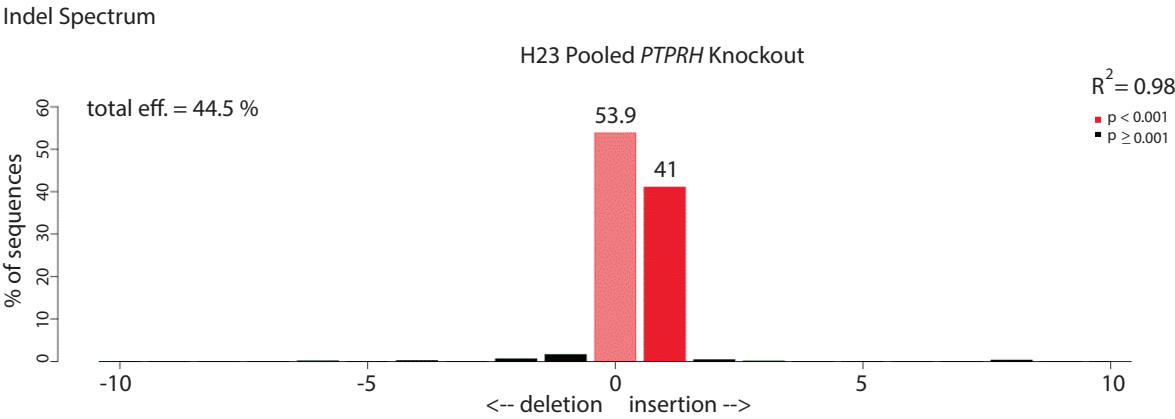

Supplement: S2 Fig — (PDF) [file pgen.1010362.s002.pdf]
